# The Effect of Processing of Hempseed on Protein Recovery and Emulsification Properties

**DOI:** 10.1155/2021/8814724

**Published:** 2021-04-13

**Authors:** Anne Pihlanto, Markus Nurmi, Nora Pap, Jarkko Mäkinen, Sari Mäkinen

**Affiliations:** Natural Resource Institute Finland, Myllytie 1, FI-31600 Jokioinen, Finland

## Abstract

The effect of carbohydrate-hydrolysing enzyme blend with or without supercritical CO_2_ (SFE) defatting on pretreat hempseed meal, hempseeds, peeled hempseeds, hempseed protein powder, and germinated hempseeds was determined. The raw materials and recovered fractions from the treatments were subjected to gel electrophoresis, and their emulsion capacity, activity, and stability as well as colour (CIE *L*∗*a*∗*b*∗ values) were determined. The highest protein contents, 65% (*w*/*w* dm), were detected in soluble fractions prepared from germinated, defatted hempseeds followed by soluble fractions of peeled, defatted hempseed, 55% (*w*/*w* dm). The gel electrophoresis showed quite similar protein profiles for all samples; however, the edestin content was lower in the germinated samples than in the others. Enzyme treatment and SFE did not have a significant effect on the emulsion properties. Germinated samples demonstrated a higher ability to stabilise emulsions (15-20%) than other pretreated samples. On the other hand, hempseed meal samples had lower emulsification activity and stability values compared to the other samples. The colour of the sample solutions varied from light to dark with a brown to yellowish colour, and PHS samples showed overall higher *L*∗ values. In conclusion, germination and peeling in combination with defatting are promising methods to produce functional protein concentrates with efficient emulsion stability and activity as well as a mild colour for food applications.

## 1. Introduction

*Cannabis sativa* L., commonly referred to as hemp, is a widely cultivated plant, providing an important source of food, fiber, and medicine [1]. The cultivation of food-grade hempseed with low *δ*-tetrahydrocannabinol (THC) has increased in Western countries (Europe and Canada). Seeds of the low-THC plants contain considerable amounts of easily digestible protein and oil, approximately 25% and over 30%, respectively [2]. Hempseed is a rich source of many interesting phytochemicals that depend on the cultivar. There is a growing interest in these compounds due to their potential health benefits [3]. Hempseed oil is rich in polyunsaturated fatty acids, especially linoleic (*ω*-6) and *α*-linolenic (*ω*-3) acids. Hempseed-derived proteins are of high quality with a superior essential amino acid composition. Unfortunately, hempseed proteins have low functional properties, especially compared to soy protein isolate [2–5] which partly prevents widespread use of hemp up-to-date. Therefore, the development of gentle and minimal processing of hempseed is essential.

Protein composition and functionality are influenced by the isolation method and purification conditions. The most widely used procedure to prepare protein isolates involves alkaline extraction/isoelectric precipitation [6, 7]. Following alkaline solubilisation of the protein components, removal of the insoluble material is carried out by centrifugation, and consequently, protein precipitation in the extracts is performed by carrying out a pH adjustment to the isoelectric point. Teh et al. [6] and Hadnađev et al. [7] reported that alkali extraction/isoelectric precipitation of hempseed proteins from hempseed meal leads to a nonappealing greenish/dark colour, mostly due to phenolics. Heating can improve solubility, but in alkali conditions (pH 12), it can lead to the formation of lysinoalanines, unless the temperature is kept under 60°C [8]. Germination is a traditional method to reduce antinutritional factors and to increase the solubility and digestibility of the legumes [9].

Enzymatic recovery of proteins is a profound technique due to its relatively gentle processing. The functional properties of the proteins can be enhanced by controlling the degree of hydrolysis in the processing [10]. On the other hand, even during enzymatic hydrolysis, protein aggregates may form and consequently impair the functional properties of hemp protein isolate such as the solubility and emulsifying and foaming properties [11, 12]. Numerous authors have investigated the possibility of enhancing the functional properties of hemp proteins. Yin et al. [11] observed that limited trypsin hydrolysis of hempseed protein isolate increased solubility but reduced the emulsification and foaming properties at the same time. Malomo and Aluko [12] improved the protein solubility and digestibility of hempseed meal with the enzymatic digestion of polysaccharide fractions, followed by protein purification with ultrafiltration processes.

The residual oil extraction conditions affect protein quality, especially the commonly used hexane extraction which may promote protein denaturation. Grijó et al. [13] investigated the applicability of pressurised n-propane for the extraction of hempseed oil from dehulled hempseed. The authors concluded that the n-propane extraction of hemp oil was an economically sound option and the oil was of good quality. Hempseed meal from cold pressing generally contains high amounts of residual oil that may enhance complex formations between lipids and proteins and thus hinder their fractionation. Defatting of the cold-pressing residue using supercritical carbon dioxide extraction (SFE) represents a gentle technology that prevents proteins from denaturing. Aladić et al. [14] compared hemp oil quality in regard to its tocopherol and pigment content and the fatty acid composition obtained by SFE, cold pressing, or n-hexane extraction. The authors concluded that when SFE was used, the oil was rich in tocopherols and low in pigments, and the fatty acid composition was not impaired in processing. Besides the oil recovery, SFE has been shown to increase the protein recovery from rapeseed [15]. Teh et al. [6] documented that the defatting process caused discolouration of the oil seed cake, while acid and alkali extractions had a significant increased and decreased redness and yellowness, respectively, of plant samples.

The objective of this research was to bring new knowledge on the effect of commercially available raw materials on the recovery and functional properties of hempseed protein. To this end, whole hempseeds, germinated hempseeds, peeled hempseeds, hempseed protein, and hempseed meal after oil recovery were used as raw materials, and comparison was made between these also after the extraction of the residual oil in the SFE process. A polysaccharide-degrading enzyme blend was utilised to enhance the recovery of soluble proteins. The recovered hempseed liquid protein extracts and remaining sediments were analysed for their basic composition, emulsification properties, and colour. Our results may provide guidelines for the utilisation of hempseed raw materials either directly or as a source of different fractions, in the production of food products like bakery products, snacks, or desserts.

## 2. Materials and Methods

### 2.1. Materials

The following hempseed (*Cannabis sativa L* cv. Finola) raw materials were used in the study: (1) hempseed meal after cold press oil recovery (SHSM) (Elixi Oil), (2) hempseed (HS) (Murtolan Hamppufarmi, Finland), (3) peeled hempseeds (PHS) (Impolan kasvitila, Sastamala), (4) hempseed protein (HP) (Impolan kasvitila, Sastamala), and (5) germinated hempseed (GHS prepared in-house from HS). Germination was performed by placing the seeds between moisturised paper layers and keeping them in the dark. After two days at room temperature, the germinated seeds were collected.

A food-grade enzyme blend of alcohol CGE (EB) was purchased from Creative Enzymes Co. (New York, USA). Alcohol CGE is a temperature stable multienzyme blend of cellulase (20,000 u/g), hemicellulose (10,000 u/g), lipase (20,000 u/g), and amylase (2,000 u/g). It is used in the food industry to reduce the viscosity of complex nonstarch carbohydrates present in cereal grains. The reagents used in electrophoresis were from Bio-Rad Laboratories Inc. (California, USA) (1610156), Sigma (St. Louis, USA) (T1503, M7154-25ML, U6504), Merck (Darmstadt, Germany) (1.13760.1000, 1.04201.1000), and GE Healthcare (Chicago, USA) (17-1312-01, 17-1311-01).

### 2.2. Enzyme-Assisted Protein Extraction

#### 2.2.1. Preliminary Tests to Increase Protein Solubility on a Small Scale

SHSM and PHS were selected for preliminary enzyme-assisted extraction tests to assess the proper solid to liquid ratio of proteins for the larger-scale study. The PHS was washed in water and soaked for 2 h in water (1 : 10). The samples were dispersed in water to obtain 2, 4, and 10% protein concentration. Consequently, the pH was adjusted to 4.5–5.5, and polysaccharide-digesting enzyme blend EB was added in a concentration of 0.5% **w**/**w** dm. Hydrolysis was performed for 4 h at 55°C under agitation after which the dispersions were suspended with a blender, and pH was adjusted to 8 using 2 M NaOH. Suspensions were filtered through a Miracloth (Merck-Millipore) to separate the solids. Liquids and sediments were stored at -20°C until analysed for protein content. The extractions were performed in triplicate for both raw materials.

#### 2.2.2. Pretreatment and Enzyme-Assisted Extraction of the Samples on a Larger Scale

**Figure 1:**
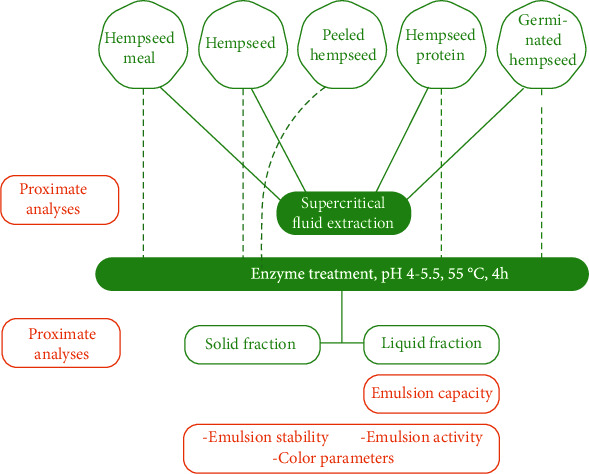
A flow chart of production solid and liquid fractions from different hempseed raw materials. Green indicates the materials and treatments used to produce solid and liquid fractions. Orange indicates the analysis methods. Dashed lines indicate the direct use of the starting material in enzymatic treatments without supercritical fluid extraction.

SHSM, HS, PHS, HP, and GHS samples were coarse milled and defatted by conducting a supercritical fluid extraction (SFE) (Figure 1) prior to enzymatic treatment. A pilot-scale SFE plant (Chematur Ecoplanning, Rauma, Finland) equipped with two extraction chambers and two separators was used. The hempseed sample mass was 2 kg in the SF-extraction. The extraction parameters for temperature and pressure were 70°C and 400 bars, respectively. The parameters were determined based on earlier experience with the pilot extraction equipment and are rather similar to those reported in the literature (40-80°C and 200-400 bars) for the supercritical extraction of hemp oil [13, 14]. The CO_2_ flow was set at 0.45 L/min, and the total extraction time was 3 hours. The defatted hempseed samples were dry milled using a 0.5 mm mesh size milling machine (Laboratory Mill 120, Perten, Italy) to distract the structure for enzymatic hydrolyses. Without defatting HS, PHS, GHS, and HSM were ground in a coarse grinder.

Hydrolysis was carried out on the milled and defatted hempseed samples on a 2.5 L scale to obtain material to analyse the emulsifying capacity, activity, and stability properties. Based on preliminary studies, a suspension with a 10% (*w*/*v*) protein concentration in water was prepared and hydrolysis was conducted as in the preliminary test (pH 4.0-5.5, 55°C, EB 0.5% *w*/*w*). After 4 h incubation, pH was adjusted to 8 to solubilise the proteins and the suspensions were filtered through Miracloth filter. The liquids and sediments were lyophilised and stored at -20°C until analyses of the proximate composition, functional properties, and colour were conducted. Control samples were prepared identically without enzymes.

### 2.3. Proximate Analyses

#### 2.3.1. Protein Quantity and Composition

In preliminary tests on the smaller scale, protein concentrations were analysed with the Bio-Rad DC™ Protein Assay using a microplate procedure and subjected to absorbance measurement at 692 nm (Thermo Scientific Multiskan EX, Ascent software). Bovine serum albumin (Sigma, A7638-1G) in ultrapure water (Milli-Q, Merck Millipore) was used as a standard. The standards were analysed in two replicates, and the samples in three replicates. Protein concentrations were calculated using a standard regression curve, and the results were expressed as means ± STD (*n* = 6).

The protein contents of the samples were determined by a Kjeltec™ 8400 analyser using the accredited Kjeldahl method according to international standards (SFS EN ISO 20483:2013, EN ISO 5983-2; Association of Official Analytical Chemists (AOAC) method 2011.11). A correction factor of 5.7 was used to express the protein contents.

An SDS-PAGE procedure was performed using 14% (*w*/*v*) acrylamide gel with 6 M urea [16] for the analyses of the composition of the proteins. The protein samples were solubilised in 0.126 M Tris-HCl, containing 0.14 M SDS, 3 *μ*M bromophenol blue, 2% (*v*/*v*) *β*-mercaptoethanol, 17.6% (*v*/*v*) glycerol, and 6 M urea. The gels were run in a 50 mM Tris buffer containing 384 mM glycine and 6.9 mM SDS. The electrophoresis was carried out using a Bio-Rad Mini-PROTEAN Tetra Cell electrophoresis dock. After electrophoresis, the gels were stained with SYPRO® Ruby Protein Gel Stain (Bio-Rad) according to the manufacturer's instructions. Relative protein quantities from the gels were analysed using ImageJ Fiji [17].

#### 2.3.2. Fat, Moisture, Ash, and Carbohydrate Content

The total fat content was determined using the SoxCap™ 2047 in combination with the Soxtec™ 2050 extraction system with a preparatory acid hydrolysis step and diethyl ether extraction (Foss A/B, Hillerød, Denmark) according to the ISO 6492 standard.

The samples were burnt for 17 hours at 500°C and at 105°C to determine the ash and the moisture content, respectively. Luke laboratories (T024) comply with standard EN ISO/IEC 17025 and are accredited by the FINAS (Finnish Accreditation Service) (Helsinki, Finland). The methods used for ash and fat composition analysis are accredited. The total carbohydrate contents were calculated using the following formula:

(1) $$\begin{array}{c} \text{Total carbohydrates }\left(\%\text{FW}\right)=100–\text{moisture }\left(\%\right)-\text{protein content }\left(\%\text{FW}\right)-\text{crude fat }\left(\%\text{FW}\right)–\text{ash }\left(\%\text{FW}\right). \end{array}$$

The total carbohydrate content was expressed as g/100 g FW.

### 2.4. Functional Properties

The emulsifying capacity, emulsion activity, and emulsion stability were determined according to the method by Satterlee et al. [18] with minor modifications, described in the paragraphs below.

For the *emulsifying capacity* (EC), samples were dissolved in water resulting in a 0.01% protein concentration, the pH was adjusted to 8 with 0.1 M NaOH, and the solutions were stirred for 30 min at room temperature (150 rpm). The samples were blended with a homogeniser (Ultraturrax), and rapeseed oil was added at a flow rate of approximately 25 mL/min. The direct-current conductivity of the emulsion was monitored with a microampere (*μ*A) meter during the emulsification. Conductivity began at 50 *μ*A, which rapidly dropped to 20 *μ*A and lower when the emulsion broke. The endpoint was determined at the moment the current dropped significantly. The amount of rapeseed oil used was recorded as the EC.

For the *emulsion activity* (*EA*) *and emulsion stability* (*ES*), hempseed samples were dissolved in water to reach a 10% (*w*/*v*) concentration. Aliquots of the suspensions, 50 mL, were taken, and the pH was adjusted to 8.0 with 0.1 M NaOH. The solution was stirred for 30 min and was diluted to 4% (*w*/*v*) with water. For the emulsion formation, 125 g of rapeseed oil was added, and the suspension was homogenised with Ultraturrax (10,000 rpm, 1 min). The dispersion was then divided into 8 parts and transferred to centrifuge tubes. Half of the samples were centrifuged immediately with 1,300 × g for 5 min in a swing-out rotor. The remaining four samples were incubated at 80°C for 30 minutes, cooled, and centrifuged as described above.

The fraction of the emulsion, dispersion, and liquid phase was measured in the centrifuge tube, and the results are expressed as

(2) $$\begin{array}{c} \text{EA}=100\times \frac{\text{the height of the emulsion}}{\text{the height of the whole dispersion}},\\ \text{ES}=100\times \frac{\text{the height of the emulsion after heat treatment}}{\text{the height of the whole dispersion after heat treatment}}. \end{array}$$

*The colour measurement* of the hempseed samples was performed using a Minolta CM-508d spectrophotometer (Mitaten Finland Ltd., Finland). The absorbance data was analysed using the SpectraMagic 1.01 software. The measurement device was first calibrated against a white calibration stone. Three parameters, *L*∗ (lightness), *a*∗ (redness), and *b*∗ (yellowness), were used to study changes in the colour. *L*∗ refers to the lightness of the samples and ranges from black = 0 to white = 100. A negative value of *a*∗ indicates green, while *a*∗ positive value indicates red. Positive *b*∗ indicates yellow, and negative *b*∗ indicates blue. The colour measurement is aimed at determining the effect of different processing steps on the discolouration of the samples.

### 2.5. Statistical Analyses

Outliers from the datasets were removed by using 1.5 times the IQR value to set limits for the acceptable values. A one-way analysis of variance was used to compare the different treatments. Analyses were performed using the SAS software.

## 3. Results and Discussion

### 3.1. Protein Recovery

The basic composition of the raw materials and defatted samples is presented in Table 1. The protein content in the raw materials varied from 24.8 (GHS) to 41.6% (HP) on a dry matter basis. Defatting with SFE increased the protein content, as expected, while the fat content decreased. After defatting, the highest protein content was 59.1% shown by the defatted PHS. In the case of SHSM, the effect of defatting on the protein content was only minor since most of the oil was already extracted in the industrial oil recovery process.

Several studies have used SFE for hempseed to produce lipid [14] cannabinoid and antioxidant fractions [19]. However, studies on the effect of SFE on protein composition are scarce. In previous studies, SFE has been reported to increase the protein content of rapeseed pressed cake from 30.5 to 34.8% [20].

#### 3.1.1. Preliminary Tests on a Small Scale to Assess the Solid to Liquid Ratio

The effect of the solid to liquid (s : l) ratio on protein solubilisation by the enzymatic hydrolysis was assessed with SHSM and PHS by testing the extraction at 2, 4, and 10% (protein *w*/*v*). Protein concentrations in the soluble fractions obtained from the extractions with the different s : l ratios and the corresponding protein yields are presented in Table 2. Soluble fractions recovered from PHS showed higher protein contents, as well as protein yields, in comparison to those of SHSM with all tested s : l ratios.

With SHSM, the s : l ratio induced only a minor effect on the protein recovery efficiency; the protein yield remained at the same level with the different s : l ratios (Table 2). In the case of PHS, the s : l ratio showed more pronounced effect on the protein yield. The higher solid contents, 4 and 10% protein (*w*/*v*), resulted in protein yields of slightly above 20% while the lowest solid content (2% protein *w*/*v*) resulted in a protein yield close to 40% (Table 2). Overall, the results indicated that protein concentrations in the soluble fractions could be increased by using a higher solid content, and sufficient protein yields can be obtained with 10% protein *w*/*v*. Therefore, 10% protein (*w*/*v*) concentration was selected for the larger-scale extraction study.

Malomo et al. [21] found that the solid content (10, 20, or 40%) did not affect the protein yields in the following isoelectric precipitation. Our results show that the effect of the solid content on protein recovery depends on the raw material type. With SHSM, the solid content could be increased from 6 to 29% without affecting the protein yield, while with PHS, increasing the solid content from 6 to 32% caused reduction of 15 percentage points in the protein yield. Solid contents of 6 to 29% for SHSM and 6 to 32% for PHS resulted in protein contents 2–10% used in the extractions. The enzyme concentration used in the experiments was 0.5% (*w*/*v*, solid) which is the highest amount suggested by the manufacturer, and therefore, we did not test higher enzyme concentrations.

#### 3.1.2. Extractions on a Larger Scale

**Figure 2:**
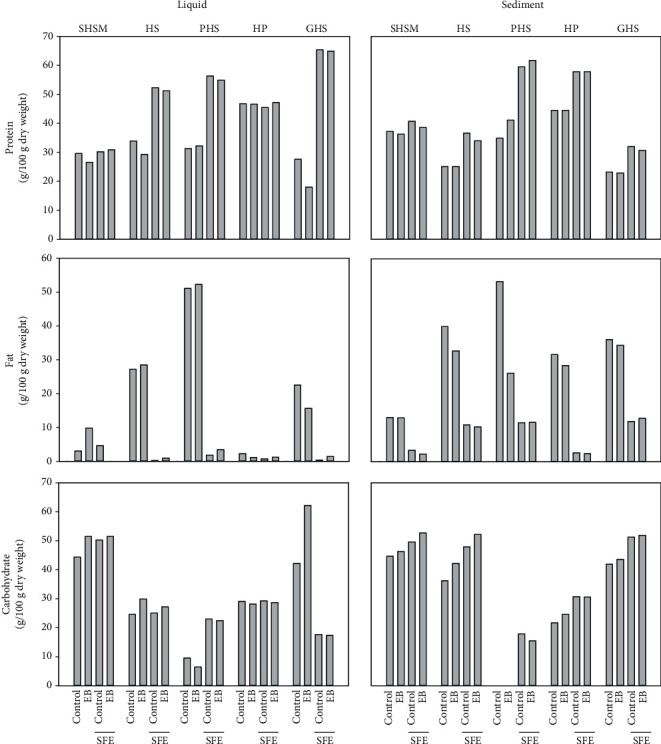
Basic composition of the raw materials before and after oil removal and enzyme treatment. Protein, fat, and carbohydrates were measured from hempseed meal after oil recovery (SHSM), hempseed (HS), peeled hempseeds (PHS), hempseed protein (HP), and germinated hempseeds (GHS) before and after an enzyme blend with alcohol CGE (EB) hydrolysis and oil removal (SFE). Measurements were done from liquid and sediment fractions of the samples. Values were calculated against dry weight.

The results from the larger scale extractions are shown in Figure 2. The protein concentration in liquid fractions varied between 18 (GHS EB) and 65.4% (GHS SFE) and in the sediment fractions between 23 (GHS control) and 61.7% (PHS SFE EB treated) (*w*/*w* %). The SFE treatment increased the protein content in liquid fractions in HS, PHS, and GHS samples, while EB treatment had only a minor effect (Figure 2). The protein yields in liquid fractions varied between 5.4 and 29.4%. The lowest yields were with SHSM samples (5.4-7.2%), and the highest were for the GHS samples (21.6-29.5%) (not shown in the figure). Earlier studies on sorghum [22] and chickpeas [23]) showed that germination increased the solubility of the proteins. Jiménez Martínez et al. [24] showed that germination of lupine seeds is a simple low-cost process that allows protein modification as germination digests the main storage proteins resulting in lower molecular weight compounds. It is suggested that germination increases the nutritional value of plant proteins due to the degradation of storage proteins, oligosaccharides, and phytate. In our present study, germination as such did not enhance the solubilisation of proteins, as the protein concentration in the liquid fractions of GHS was lower in comparison to the other samples (Figure 2). However, when germination was followed by SFE, the protein solubilisation was increased efficiently as the protein contents in the GHS liquid fractions increased from 25% to over 60% when SFE was applied.

It is known that some carbohydrate degrading enzymes alone or in combination with proteases can be used to extract proteins from rapeseed meals [25]. Up to 83% of the total protein has been extracted from dehulled, cold-pressed rapeseed pressed cake using hemicellulases, pectinases, and cellulases in combination with protease treatment [26]. Rommi et al. [15] found higher protein recovery after carbohydrate-hydrolysing enzyme treatment of the pressed cake, but the extent to which the enzyme treatment enhanced the protein yield depended considerably on the extraction conditions. Malomo and Aluko [12] used an enzyme mixture with cellulase, hemicellulose, zylanase, and phytase followed by ultrafiltration to remove polysaccharide fragments. The final product had twice the original protein content of the starting material.

**Figure 3:**
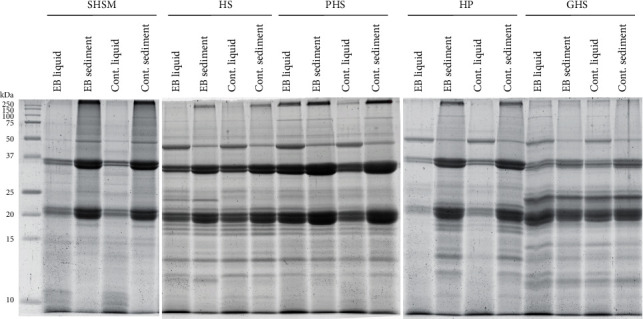
Protein profiles of hemp raw materials before and after enzyme treatment. SDS-PAGE was performed to compare protein profiles between raw materials, hempseed meal after oil recovery (SHSM), hempseed (HS), peeled hempseeds (PHS), hempseed protein (HP), and germinated hempseeds (GHS) before and after an enzyme blend for alcohol CGE (EB) treatment. Protein profiles were analysed from both the liquid and sediment fraction of the sample.

The protein profiles of the soluble fractions and sediments of SHSM, HS, PHS, HP, and GHS were analysed using SDS-PAGE (Figure 3). The SDS profiles show that the main polypeptides were 35 and 22 kDa (edestin). Protein band intensities were quantified by using Fiji ImageJ software. The amount of edestin compared to total proteins detected in the gel was less in all GHS samples except the EB-treated liquid fraction where the edestin/total protein ratio was like the HS and HP samples. In general, the amount of edestin was 10-20% less in GHS than other starting material. The GHS samples did not contain a high molecular weight in protein compared to the starting material. This means that the storage proteins (edestin) were partly degraded, and therefore, the protein solubility increased.

#### 3.1.3. Carbohydrate and Fat Contents after Processing

Table 1 shows the carbohydrate content of the raw materials and the effect of the defatting process. The carbohydrate content of the raw materials varied significantly; the lowest carbohydrate content was shown in peeled samples before and after defatting, 7.7% and 12.4%, respectively. This was as expected, since peeling removes the carbohydrates that are present mainly in the outer part of the seeds. The highest carbohydrate contents were shown by SHSM, 49.3 and 43.5%, before and after defatting, respectively. There are indications that the germination of legumes can reduce the oligosaccharide content [27]. This seems not to be the case with the hempseeds as the carbohydrate content in GHS was at the same level as the HS (Table 1). In the liquid fractions, the lowest carbohydrate contents were detected in PHS-derived samples (Figure 2), which is in line with the low carbohydrate content of PHS as a raw material. Among the liquid fractions, the SHSM-derived fractions had the highest carbohydrate contents together with EB-treated GHS (Figure 2). Interestingly, the carbohydrate content in the liquid GHS samples was much lower after SFE treatment (17%). This was not the case for the other raw materials tested, since SFE treatment increased the carbohydrate content in PHS samples and had no effect on other raw materials. EB treatment did not induce any significant effects on the carbohydrate contents.

Carbohydrates are common constituents in food, serving as a nutritious source of energy and dietary fiber [28]. Hempseed carbohydrates contribute to sensory properties, especially the texture and sweetness of food products [29]. Depending on the structure, carbohydrates interact with the other compounds in the food matrix and can affect greatly the functional properties [30].

The fat content of the raw materials varied from 1.9% (defatted HP) to 49.4% (HP). The PHS also displayed a high fat content, 48.5%, while the lowest fat content was observed for SHSM (Table 1). This was as expected since most of the fat had been removed from SHSM already in the industrial oil pressing process. In the liquid and sediment fractions, the fat content varied between 0 and 52% of the dm (Figure 2). In general, sediment fractions had a higher fat content in comparison to the soluble fractions. The lowest fat contents were observed in the soluble fractions treated with SFE with or without enzyme. However, the fat contents in sediment fractions prepared from SFE-treated hempseed materials varied from 2 to 10% of dm. When SFE was not applied, the fat content varied from 1% (liquid HP EB) to 51% of the dm (liquid PHS EB and PHS sediment). Fat levels are known to contribute to functional properties, such as emulsion stability, texture, colour, and sensory characteristics [31].

### 3.2. Functional Properties

#### 3.2.1. Emulsion Properties

The emulsifying activity of protein lies in its potential to interact with and stabilise an oil-water mixture to prevent phase separation. The exposure of hydrophobic amino acid residues would enhance the formation of good emulsions because of the propensity to interact with the hydrophobic liquid phase. Emulsion capacity (EC) measures the ability of soluble proteins to migrate to the water-oil interface, indicating that solubility and conformation of proteins are affected by environmental conditions, such as ionic strength and pH. Other parameters, such as emulsion activity (EA) and emulsion stability (ES), should be considered to measure the ability of proteins to form an emulsion [18].

**Figure 4:**
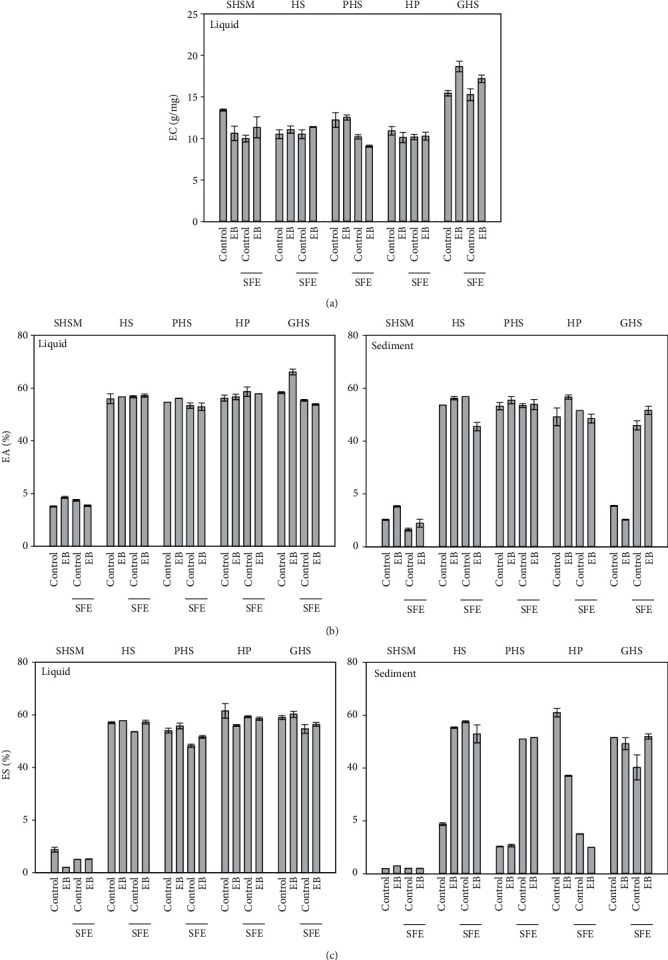
Emulsion properties of raw materials before and after oil removal and enzyme treatment. (a) Emulsion capacity (EC), (b) emulsion activity (EA), and (c) emulsion stability (ES) were measured from hempseed meal after oil recovery (SHSM), hempseed (HS), peeled hempseeds (PHS), hempseed protein (HP), and germinated hempseeds (GHS) before and after an enzyme blend for alcohol CGE (EB) hydrolysis and oil removal (SFE). Measurements were taken from liquid and sediment fractions of the sample.

*(1) Emulsion Capacity*.  Figure 4(a) shows the EC of the liquid fractions after enzymatic treatments and the respective control samples. No data is shown for the sediment fractions since the low solubility of the sediments impeded reliable EC measurement values. The highest EC values were observed in the liquid fractions of GHS (EC 15.5-18.7 g/mg) whereas for other samples, the EC values varied from 9.2 to 13.4 g/mg.

Enzymatic treatment without defatting only increased the EC for the GHS samples (21%; *p* < 0.001). In the case of the SHSM, the control sample showed superior EC compared to the enzyme-treated sample (*p* < 0.009). Defatting reduced the ECs of SHSM and PHS significantly compared to controls (*p* < 0.01, *p* < 0.005). However, defatting did not affect the ECs of HS, HP, and GHS (*p* = 0.22-0.49). Making similar comparisons for the defatted and enzyme-treated materials, no significant difference in ECs between the defatted control and defatted enzyme treatment for SHSM HP and HS was found. However, enzyme treatment increased the EC significantly in GHS (*p* = 0.011) defatted samples.

*(2) Emulsion Activity*. EA values were measured for all liquid fractions and sediments. Overall, the EAs of the liquids and sediments varied from 6 (SFE-treated SHSM sediments) to 66% (EB-treated GHS liquid) (Figure 4(b)). The lowest values were found in SHSM sediments and GHS sediments without SFE treatment. The highest value was found in the GHS liquid fraction after EB treatment. The EAs of SHSM liquid fractions were low overall (15-20%) in comparison to the liquid fractions of the other samples (EAs 53-66%) (*p* < 0.01). Enzyme treatment had little effect on the EA values in the liquid fraction. The only exception was GHS where the EB treatment increased the EA (13%; *p* < 0.001). Sediment fractions showed EA values at the same level as the liquid fractions with some exceptions. SHSM sediments had 6-10% lower EA values than the corresponding liquid fractions. GHS fraction sediments without defatting had much lower EAs (10% and 16%) than the respective liquid fractions (58% and 66%). In the sediment fraction, defatting greatly increased the EA of the GHS samples (30% and 42%; *p* < 0.001). The EB treatment after defatting decreased the EA of HS (56% and 46%; *p* < 0.001).

*(3) Emulsion Stability*. The ES values of liquid fractions varied from 2 (EB-treated SHSM) to 62% (HP) (Figure 4(c)). Most of the liquid fractions showed ES values between 50 and 60%, but the SHSM had remarkably lower ES values (2-9%). Neither the enzyme treatment nor defatting had an impact on the stability. The maximum variation was only 6% between SFE and nontreated samples.

In the sediment samples, the ES varied from 2 (SHSM) to 61% (HP). Overall, the lowest values were observed for the SHSM samples. Defatting significantly reduced the ES of HP (*p* < 0.01) but increased in PHS. For the other raw materials, defatting did not induce any significant effects. Enzymatic treatment increased the stability of the HS from 19 to 55 ES %, but this decreased in HP from 61 to 37 ES % (*p* < 0.001). The increase of the ES was also seen with the GHS-derived sediment fractions (from 40 to 52 ES %); however, the effect was dependent on the defatting of the raw material (Figure 4(c)).

Various synthetic emulsifiers are used in the food industry to facilitate emulsion formation and enhance emulsion stability. Casein, whey, and soy isolates are the most commonly used protein-based emulsifiers, but there is a need for alternative plant protein-based emulsifiers [32]. Lupine [33] and peas [34] have shown promising emulsification properties, but to our knowledge, data on the emulsification properties of hempseed is scarce. Tang et al. [5] tested EA and ES at pH values 3-8 and noticed that the values for hempseed protein isolates had a significantly lower EA than for soy protein isolates. Moreover, they found that EA profiles were associated with solubility values. Protein-polysaccharide complexes are also known to stabilise the emulsions [35]. Germination changes the carbohydrate and protein composition which may result in increased interactions between proteins and carbohydrates. Thus, the observed increase in the emulsion capacity activities in germination is presumably induced by the changes in carbohydrate and protein composition. In addition to solubility, other parameters, such as the surface hydrophobicity and aggregation state of proteins, affect emulsion activity. Conformational flexibility has also been suggested to be a critical factor for the emulsion activity of proteins, especially in the case of soy and milk proteins [36]. It is challenging to compare the emulsion activity and stability results obtained in this study to earlier reported ones due to different methods and how the results are presented. In the present study, the EA and ES values of the proteinaceous liquid fractions of hempseed are comparable to the values reported for milk whey (ES 77-86%) [36] and values from commercial hempseed protein concentrate [66].

One of the major challenges in developing sustainable and natural plant protein-based emulsifiers is the establishment of effective processing methods for protein isolation and fractionation. The present study showed that germination with consequent SFE treatment and protein extraction can be applied to produce protein-rich fractions with good emulsification properties.

#### 3.2.2. Colour Parameters

One of the most important issues to overcome the utilisation of by-products or derived isolates in model foods is the colour of the ingredient. A darkish green-grey colour that might be formed during hemp processing needs to be avoided, since this colour is not very appealing. To permit the use of hemp hydrolysate products, light-coloured fractions are desirable instead of the typical dark green fractions. In this research, the colour attribute changes during the 2-step processing were followed, and the *L*∗*a*∗*b*∗ values were measured before and after the defatting and after the hydrolysis process.

Table 3 shows that the colour changed during processing. When comparing the lightness of the liquid fractions of different raw materials, PHS shows overall higher values of *L*∗. This is expected since peeling removes the dark surface of the seed. SFE treatment increased the lightness of HS and GHS samples. The EB treatment-induced variation in the *L*∗ values depend on the starting material. However, no major effect was seen in any samples. Sediment fractions were overall darker compared to the liquid fractions. Similarly, with the liquid fractions, the PHS sediment was lighter in colour compared to other sediment samples. SFE clearly improved the colour in all samples except PHS. Additionally, in PHS treated with EB, fat removal increased the *L*∗ value. The redness value (*a*∗) varied between -0.9 (PHS control liquid) and 5.9 (GHS EB-treated liquid). The yellowness (*b*∗) varied from 11.1 (GHS SFE solid) to 30.6 for the SHSM control liquid fraction. These range values indicate that the colour of the sample solutions varied from light to dark with a brown to yellowish colour. Overall, the sediment fractions were more brownish in colour compared to the liquid fractions.

The lightness has been attributed to the difference in the light scattering effect by particles of different sizes. The smallest particles with an increased surface area scatter more light and appear to be lighter unlike the fractions with larger particle size [37]. Pojić et al. [38] noticed that hemp meal's finest fractions had the highest lightness. Often, plant products have a greenish colour that does not meet consumer preferences, but usually, light yellow is well accepted. In this study, it was noticed that the liquid PHS sample after SFE and EB treatment was most acceptable in colour.

## 4. Conclusions

The finding of this study showed that besides the nutritional role of hempseed protein, there is potential for use as a functional ingredient in the food industry. The results showed that germination and peeling combined with defatting enhanced protein solubility and resulted in a mild colour and efficient emulsification properties. These processes can be utilised in different food processing applications to produce products such as plant-based drinks from hempseeds. Germination and peeling are usually considered economically feasible, mild, and environmentally friendly processes.

## Figures and Tables

**Table 1:** Basic composition of raw materials and defatted samples.

Sample	Protein (g/100 g)	Fat (oil) (g/100 g)	Carbohydrates (g/100 g)	Moisture (g/100 g)
SHSM	34.6	11.1	49.3	4.93
SHSM, defatted	36.9	2.0	43.5	4.5
HS	25.1	23.1	38.5	7.56
HS, defatted	35.9	8.56	43.8	4.00
PHS	31.0	48.5	7.7	7.07
PHS, defatted	59.1	8.29	12.4	8.38
HP	41.6	49.4	19.5	8.56
HP, defatted	54.3	1.87	27.6	5.05
GHS	24.8	30.7	36.3	1.69^a)^
GHS, defatted	37.1	8.17	42.6	3.85

^a^The germinated sample was freeze-dried before defatting due to the risk of spoilage.

**Table 2:** Protein concentrations of the soluble fractions and corresponding protein yields obtained from the extractions using different solid contents.

	Protein content in the extracts (mg/ml)			Protein yield (%)		
	2% extraction	4% extraction	10% extraction	2% extraction	4% extraction	10% extraction
SHSM	2.6 ± 0.2	4.0 ± 0.3	8.7 ± 0.6	13.9 ± 0.8	11.7 ± 0.8	12.1 ± 0.8
PHS	7.5 ± 0.8	8.2 ± 1.2	19.3 ± 2.5	37.2 ± 3.6	21.0 ± 2.8	22.0 ± 2.3

**Table 3:** Colour profiles of the processed hempseed materials. The colour profiles were measured from hempseed samples using the *L*∗*a*∗*b* colour space.

	Liquid			Sediment		
	*L*∗	*a*∗	*b*∗	*L*∗	*a*∗	*b*∗
SHSM	58.038	0.795	30.615	25.915	3.057	16.553
SHSM EB	45.867	-0.234	25.966	25.0233	2.303	13.856
SHSM SFE	57.485	0.392	25.257	36.8466	3.404	16.368
SHSM SFE EB	58.213	2.163	22.477	37.5702	4.132	15.595
HS	38.115	2.774	22.288	13.607	2.518	11.256
HS EB	48.301	4.811	29.465	21.691	3.146	13.911
HS SFE	73.184	2.902	21.992	34.735	3.272	14.381
HS SFE EB	60.315	2.694	19.1042	37.669	3.628	16.401
PHS	78.388	-0.902	18.975	39.007	0.289	17.092
PHS EB	66.737	-0.598	18.483	36.181	0.188	16.480
PHS SFE	69.298	-0.602	16.604	40.801	0.761	13.764
PHS SFE EB	79.249	-0.652	18.129	53.179	0.466	15.691
HP	66.047	-0.329	22.324	28.121	3.559	21.999
PHS EB	61.385	-0.118	21.713	25.897	3.077	20.303
PHS SFE	68.205	-0.239	15.124	42.4	3.067	17.547
PHS SFE EB	52.654	0.809	14.022	43.141	3.186	17.328
GHS	41.013	5.185	27.132	13.936	2.923	12.879
GHS EB	47.128	5.949	27.791	12.372	3.509	11.626
GHS SFE	58.091	2.561	20.807	37.821	3.598	15.849
GHS SFE EB	62.503	1.398	19.777	26.944	2.827	12.619

## Data Availability

All data generated or analysed during this study are included in this published article.
